# Effect sizes of *APOE* e4 on the same general cognitive ability test taken by the same people from age 11 to age 90: The Lothian Birth Cohorts 1921 and 1936

**DOI:** 10.21203/rs.3.rs-6462650/v1

**Published:** 2025-05-07

**Authors:** Ian Deary, Sarah Harris, Tom Russ, Simon Cox, Janie Corley

**Affiliations:** University of Edinburgh; The University of Edinburgh; University of Edinburgh; University of Edinburgh; University of Edinburgh

## Abstract

Variation in the gene for apolipoprotein E (*APOE)* is one of the few variables that is associated with individual differences in age-related cognitive decline in humans. Therefore, it is important to understand the conditions that affect the strength of its effect. Here we examine how the effect size of *APOE* variation (possession of one or more e4 alleles) on a test of general cognitive ability changes with age from 11 to 90 years. The data are from the Lothian Birth Cohorts of 1936 and 1921 who took the same cognitive test (the Moray House Test) at, respectively, 11, 70, 73, 79, and 11, 79, 87, 90. The standardised absolute effect of *APOE* e4 on general cognitive ability was about zero at ages 11 (beta < 0.05) and 70 (beta ≤ 0.025) and increased linearly to beta = 0.30 (p < 0.001) at age 90. The effect sizes were minimally affected by adjusting for medical conditions (hypertension, diabetes, cardiovascular disease, stroke). However, the results were less robust to removing those participants who had developed dementia; effect sizes were reduced by about a third to a half, and were largely non-significant. The results suggest that the negative effect of *APOE* e4 on cognitive functioning becomes greater with age; this urges more work to understand the mechanisms by which e4 status renders the older person’s brain increasingly vulnerable to cognitive decline and dementia.

## Introduction

As an outcome variable, cognitive functioning is a moving target. In humans, aspects of cognitive capability show mean declines after young adulthood (1, 2). Older people score lower on some tests of, for example, processing speed, memory, and reasoning, whereas verbal capability, general knowledge, and some numerical abilities age better (3, 4). To complicate the picture, people show individual differences in cognitive test scores throughout the life course and, although these differences are moderately stable across several decades (5–8), there are individual differences in the changes that occur between youth and older age (2, 9, 10). All of this matters. Even when assessed in childhood or youth, cognitive ability relates to later life outcomes: it predicts how well people perform in educational and occupational settings (11), and higher cognitive ability in youth is related to future better health and longer life (12). Given this, it is predictable and empirically demonstrated that higher cognitive function in adulthood and older age is related to better management of daily living and a higher likelihood of remaining independent (13, 14). It follows that it is interesting and practically important to discover the determinants of people’s differences in cognitive functioning. And a moment’s reflection reveals that that is at least two questions. First, what variables are related to cognitive differences at any given age (and we note that a variable related to cognitive functioning at one age might not be associated at some other age)? Second, what variables are related to individual differences in changes from a given age to a later age? Of course, that depends on the stage of the life course that is being investigated; i.e., it is of interest to And out what predicts improvements through childhood to adulthood, what predicts the declines from youth to older age, and what predicts declines within older ages (7).

Focussing on older age, there is much published work that tries to find the variables that are associated with healthy cognitive ageing, i.e., what predicts less decline—or even some improvement—from a given age to a later age (15–17). (Of course, the special hope is to discover modifiable associates, so that people’s cognitive trajectories can be made to proceed less steeply downward.) The list of candidate predictors is long, taking in genetics, demographics, physical and mental health, fitness, personality, lifestyle, diet, physical and social environment, and so forth. Popular media, books, and sundry sources of advice beyond the academic journals abound with ideas to keep one’s thinking sharp (18–21). Though this looks encouraging at a distance, the reality is more stark. There are few replicated determinants of people’s differences age-related cognitive changes (15, 16, 22). Most effect sizes are small. Some so-called associates of cognitive ageing are either outcomes rather than causes of cognitive capability, or cognition and the putative predictor are both related to some prior confounder (15, 23). In a stringent test, when many putative predictors of cognitive ageing are entered together in a multivariate setting, there are few significant results (16).

The equation that tries to solve the problem of what contributes to cognitive ageing differences is simple; i.e., beyond the level of cognitive ability that each person had on a previous occasion, what predicts their cognitive score on subsequent occasions? To be clear, it is important that the predictor is set as an exposure to the outcome of cognitive change, not just a one-time cognitive test score. In older age—the 70s, for example—cognitive test scores from adolescence account for about half of the variance in cognitive ability. Beyond prior cognitive ability (6, 7), one variable that stands out as a frequently-reported additional predictor of cognitive ability test scores in older age is possession of the e4 allele of the gene for apolipoprotein E *(APOE)* (16, 24). The approximately quarter of the population who have at least one copy of the *APOE* e4 allele are more likely to develop dementia and also to have more age-related cognitive decline that is short of dementia than those who don’t possess it (24–26). There are studies demonstrating that variation in *APOE* genotype is associated with cognitive function in older age, cognitive change from childhood to older age (27), and cognitive change within older age (16, 28). Few studies in the field are directly comparable, owing to differences including the age-range studied, the cognitive tests used, and the categories of *APOE* genotype that are used. The Whitehall II study’s examination of *APOE’s* association with global cognitive function from age 45 to 85 across five waves of testing is comparable in its concerns to the present study (29). They found that *APOE* e4 heterozygotes had poorer cognitive function and steeper cognitive decline from age 75 onwards, compared to non-APOE carriers, with no differences between 60 and 70 years. Similarly, in the Chicago Health and Ageing Project, those who were aged about 84 years and possessed e3e4 *APOE* genotypes declined twice as much (about 1 versus 0.5 standard deviations) in global cognitive function over ten years than those with e3e3 (30). No such differences in decline were found in those aged about 66 years.

The need for more research was the conclusion of a review that assembled results from 65 cross-sectional and 46 longitudinal studies of the association between *APOE* variants and cognitive test scores in people without cognitive impairment or dementia (24). The reviewed studies were published between 1994 and 2017. The results were presented across a number of cognitive phenotypes and constructs. Neither the cross-sectional results nor the longitudinal results were consistent. Sometimes people with the e4 allele of APOEhad lower cross-sectional/baseline test scores and sometimes not; ditto with accelerated cognitive change. There was an indication that episodic memory might be particularly affected by the e4 allele. The authors suggested that any detrimental effects of *APOE* e4 on cognitive function might be less in the oldest old. They recognised that a meta-analytic review had found overall detrimental effects of e4 allele possession on global cognition, episodic memory, executive function, and perceptual speed (31). They also discussed methodological challenges at length, aiming to encourage studies that might be more informative. Recommendations included having a large (e.g., > 1000) sample size, starting with a sample that is older than their 60s, conducting longitudinal research with a sufficient follow-up period, trying to rule out prodromal dementia among apparently healthy people, using sensitive rather than brief cognitive tests, and using one or a small number of cognitive phenotypes that are affected by ageing and dementia to minimise multiple comparisons.

The present study accords with the above recommendations. We declare that, among the *APOE*-cognition studies mentioned heretofore, there are several that employed data from the Lothian Birth Cohorts whose data are used in the present study (16, 27, 32–37). Only one study of *APOE* variants in the Lothian Birth Cohorts reported on the cognitive phenotype that is used in the present study (the Moray House Test) and that included only two time points—age 11 and age 79—in the Lothian Birth Cohort 1921 (27). Moreover, the present study is the first to include all testing waves to date of both cohorts in the same study. Notably, the younger (LBC1936) and older (LBC1921) cohorts’ data collection has proceeded substantially since that publication; the two cohorts overlap almost perfectly in sample size at age 79, allowing a two-cohort, uninterrupted follow-up from age 70 to age 90.

The present study addresses a specific question that has not been addressed to date, i.e., what is the effect size of the e4 allele of *APOE* on general cognitive ability on the same people taking the same cognitive test from age 11 to age 90?

## Materials and Methods

### Participants

The Lothian Birth Cohorts of 1921 and 1936 (LBC1921 and LBC1936) are two longitudinal studies based in Scotland, investigating cognitive ageing and health trajectories across the lifespan (10, 16, 38, 39). Most of the participants in these cohorts took part in the Scottish Mental Surveys of 1932 (40) or 1947 (41), respectively, which tested the intelligence of nearly all Scottish school children born in 1921 and 1936, using a validated IQ-type test—The Moray House Test No. 12 (MHT). These early-life assessments provide rarely-available baseline data for studying cognitive ageing.

The LBC1921 recruited 550 participants at baseline in older age (age ~ 79), with follow-ups until age 90. The LBC1936 recruited 1,091 participants at baseline in older age (age ~ 70) and continues ongoing follow-ups at the time of writing (2025). Both cohorts provide rich longitudinal data, including cognitive tests, health measures, genetic profiles, and psycho-social and sociodemographic variables (39).

**Figure 1:**
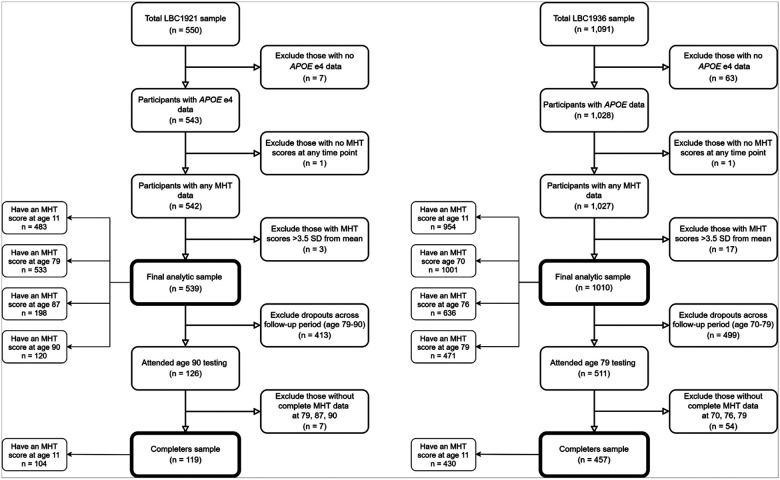
Flowcharts of the LBC1921 and LBC1936 analytic samples.

For the current study, ‘All comers’ included participants with *APOE* e4 and Moray House Test data from at least one time point (LBC1921: n = 457; LBC1936: n = 1,010). ‘Completers’ were those who completed the MHT at all three later-life waves (LBC1921: ages 79, 87, 90, n = 119; LBC1936: ages 70, 76, 79, n = 457). Participant flowcharts are presented in Fig. 1.

All participants provided written informed consent. Ethical approval for the LBC1921 study was provided by the Lothian Research Ethics Committee for test waves 1–3 at ages 79, 83 and 87 (LREC/1998/4/183, LREC/2003/7/23, 1702/98/4/183) and the Scotland A Research Ethics Committee for test wave 4 at age 90 (10/MRE00/87, 10/MRE00/87). Ethical approval for the LBC1936 study was obtained from the Multicentre Research Ethics Committee for Scotland (baseline, MREC/01/0/56), the Lothian Research Ethics Committee (age 70, LREC/2003/2/29), and the Scotland A Research Ethics Committee (ages 73, 76, 79, 82, 07/MRE00/58).

#### APOE e4 status

*APOE* e4 carrier status (no = 0, yes = 1) was determined through genotyping of DNA extracted from blood or saliva samples. Genotyping focused on two key single nucleotide polymorphisms (SNPs), rs429358 and rs7412, which define the e2, e3, and e4 alleles of the *APOE* gene. *APOE* was genotyped in LBC1921 by polymerase chain amplification restriction fragment length polymorphism (PCR-RFLP) analysis as described in (33). Genotyping of LBC1936 was conducted by the Genetics Core at the Wellcome Trust Clinical Research Facility at the Western General, Edinburgh using TaqMan technology (34).

### The Moray House Test (MHT)

The MHT (No. 12, revised) is a group-administered cognitive test originally developed to measure general intelligence in Scottish school children. It was administered to almost all 1921-born and 1936-born children in Scotland as part of the Scottish Mental Surveys of 1932 (40) and 1947 (41), respectively, under standardised conditions. Most of the Lothian Birth Cohorts’ participants—those born in 1921 and 1936—completed the MHT at age about 11 years, providing a childhood measure of cognitive ability that serves as a baseline in the lifelong study of cognitive ageing. These data, archived by the Scottish Council for Research in Education were made available for the LBC studies. The MHT includes a variety of items assessing verbal reasoning (which predominates), spatial awareness, arithmetic, and other cognitive skills. Across the life course, MHT scores are highly correlated with concurrently-administered measures of general intelligence *(g* factor), making it a validated measure of childhood and older-age cognitive ability (40, 42). The same MHT test was re-administered to Lothian Birth Cohorts participants in older age, at study baseline and two further later-life waves (see above), with identical wording, instructions, and time limits. For the present study, raw MHT score (maximum = 76) served as the dependent variable.

### Covariates

In the LBC1921 and LBC1936, several demographic and health-related variables were assessed at each wave of testing. Age (calculated in days), sex (male = 1, female = 2), and health variables (self-reported history of cardiovascular disease, stroke, hypertension, and diabetes) were included as covariates.

Participants were free from dementia at recruitment and subsequent presence of dementia was ascertained using medical consensus based on health records and some in-person visits (37, 43–45). A similar multi-source approach was used in both cohorts: electronic medical records were examined in detail for all consenting participants by a team of clinicians; dementia recorded as a cause of death on death certificates (in any position) was noted; and a subset of participants were visited at home for an assessment by a research doctor if they expressed concerns about their memory at any wave of follow up or if the research team judged that there were reasons to be concerned about cognitive decline. Separate multi-specialty consensus meetings were held for each cohort where each individual’s dementia status (probable, possible, or no dementia) was agreed, along with subtype where it was possible to identify this (again, probable or possible, depending on the amount of information available). In LBC1921 and LBC1936, respectively, 117/527 (in December 2016) and 125/865 (in August 2022) individuals were identified as having probable (110 and 118, respectively) or possible dementia. Dementia ascertainment was used in sensitivity analyses (see below).

### Statistical Analysis

Two sets of statistical analyses tested two related, but slightly different questions.

First, we used linear regression simply to ask whether there was a widening of any cognitive gap between *APOE* e4 carriers vs non-carriers as people grew older. Thus, we conducted separate regression analyses for each testing instance (wave). Specifically, = 0.33), which accords with the cross-sectional associations between *APOE* e4 status and MHT score in childhood (age 11) and in later life (LBC1936: ages 70, 76, 79; LBC1921: ages 79, 87, 90). Model 1 adjusted for age and sex, whereas model 2 included additional adjustments for health variables (CVD, stroke, hypertension, and diabetes) in the later-life data collection waves. Analyses were stratified by cohort and by subgroup (‘all comers’ and ‘completers’). A meta-analytic estimate for MHT scores at age 79 combined results across both the LBC1921 and LBC1936 because both cohorts had been tested at that age. Sensitivity analyses excluded participants with dementia diagnosed post-baseline (both cohorts were dementia-free at baseline), up to the end of the dementia ascertainment follow-up period (which was completed after the data analysed in the present study were collected).

Second, we asked, using the same data but this time applying a longitudinal analytical approach, whether possession of *APOE* e4 was associated with steeper declines in MHT score in older age. In a structural equation modelling setting, latent growth curve models estimated individuals’ trajectories of MHT scores over time, focussing on the association between *APOE* e4 carrier status and cognitive performance in older age. These models provide estimates for the intercept (baseline cognitive ability) and the slope (rate of cognitive change over time), with adjustments for covariates, to test whether *APOE* e4 carriers differ in their initial cognitive performance and/or experience a steeper decline compared to non-carriers. The structural equation modelling-based approach was consistent with previous work on the Lothian Birth Cohorts (16, 46, 47). Path weights corresponded to average time lags between waves: LBC1936 (0, 6.75, 9.81 years) and LBC1921 (0, 7.53, 11.04 years).

Linear regression and growth curve models were conducted in R (v4.3.3, “Tidyverse Teachings”, R Core Team, Vienna, Austria), using the ‘lavaan’ package (48), with standardised estimates, p-values, and confidence intervals reported. Model fit for growth curve modelling was tested using absolute fit indices: Root Mean Square Error of Approximation (RMSEA; values < 0.06 considered acceptable), Comparative Fit Index (CFI; values > 0.95 considered acceptable), and Standardized Root Mean Square Residual (SRMR; values < 0.08 considered acceptable).

Principal component analysis was used in SPSS version 29 to examine the loadings of the Moray House Test in the setting of the other 13 varied cognitive tests used in the first testing wave (age 70) of the Lothian Birth Cohort 1936 (49). Owing to their high correlation, the National Adult Reading Test and the Wechsler Test of Adult Reading (which share the same maximum score) were averaged to form a single indicator of verbal ability. Component number was determined using the eigenvalues > 1 criterion and scree slope inspection. This analysis was carried out to offer more information about the Moray House Test as an indicator of general cognitive functioning.

## Results

Considering the all comers of both cohorts, 30% of the LBC1936 had the e4 allele of *APOE* as did 27% of the LBC1921 (Table 1). A sizeable minority of both cohorts reported hypertension and/or cardiovascular disease with much smaller minorities reporting diabetes or stroke (Table 1).

### The Moray House Test scores from age 11 to age 90

Mean Moray House Test scores for both LBC1921 and LBC1936 were always higher in older ages than at age 11 years (Table 2). Within older age, the MHT scores declined for both cohorts whether one considers the all comers or the completers. The LBC1936 had a higher mean score on MHT at age 11 than the LBC1921. The difference is 2.7 points, roughly reflecting the whole-population difference found between the Mental Surveys of 1932 and 1947 (41). Both cohorts were tested at the same older age—79 years—at which time the MHT score difference was similar to that at age 11, i.e., 3.2 points; we note that this was the third exposure to this test within older age for the LBC1936 but only the first for the LBC1921. Considering completers only, the mean decline in LBC1936 from age 70 to age 79 was 0.44 SD (0.048 SD per year), and for LBC1921 from age 79 to age 90 was 0.98 SD (0.089 SD per year).

**Figure 2:**
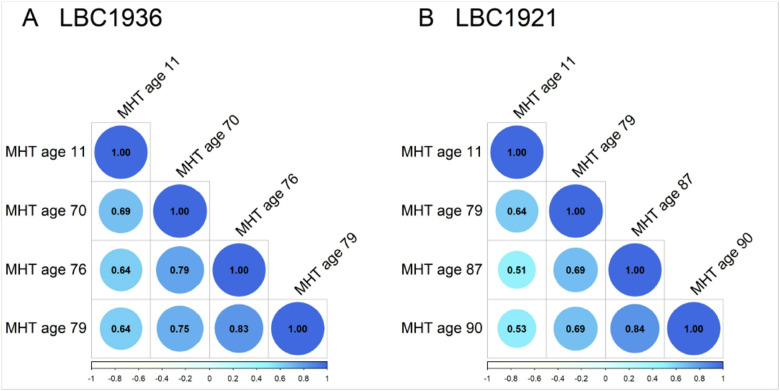
Correlation plots of all Moray House Test scores at each age point (see Table 2). Colour and size of circle indicate the magnitude of correlation between the MHT scores. All correlations are significant at the P < 0.01 level. For standard errors see Supplementary Table S1.

The stability coefficients for the Moray House Test are shown in Fig. 2 (and Supplementary Table 1). Some of these have been published by us previously (6). The age 11 versus age 79 association—the only comparison that is at the same ages for the two Lothian Birth Cohorts—is 0.64 for both LBC1921 and LBC1936. Correlations within older age are high, i.e., almost at or well above 0.7.

### Linear regression: the APOE e4 effect sizes on Moray House Test scores at each testing occasion from age 11 to age 90

**Figure 3:**
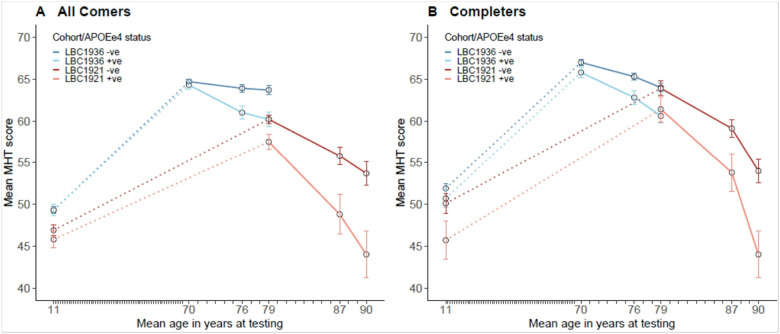
Mean Moray House Test (MHT) scores by *APOE* e4 status at age 11 and at each wave of testing across older age for the LBC1936 and LBC1921 for all comers (left) and for completers only (right) (see Table 2). Dotted lines denote the change in MHT score from age 11 to the first MHT score in older age. A continuous line denotes the change in MHT score across older age. The ticks representing individual years between childhood (age 11) and age 70 have been compressed for visual clarity. Error bars represent standard errors.

For descriptive purposes we show the mean Moray House Test scores for the LBC1921 and LBC1936 split by whether or not people possess the e4 allele of *APOE* (Table 2, Fig. 3). This is done for all comers and completers. There is little difference in the scores of e4- and e4 + groups at age 11 and age 70. Thereafter, the e4 + group appears to decline more steeply than the e4- group; mostly, the differences between e4- and e4 + scores are greater at older ages. The completers’ panel of Fig. 3 shows that the LBC1921 and LBC1936 have similar values at age 79 with the e4 + group scoring lower in both cohorts. Overall—including both cohorts—the mean decline in Moray House Test score from age 70 to age 90 for all comers is 20.3 points (21.8 for completers) for those with an *APOE* e4 allele and 11.0 points (12.9 for completers) for those without one.

**Figure 4:**
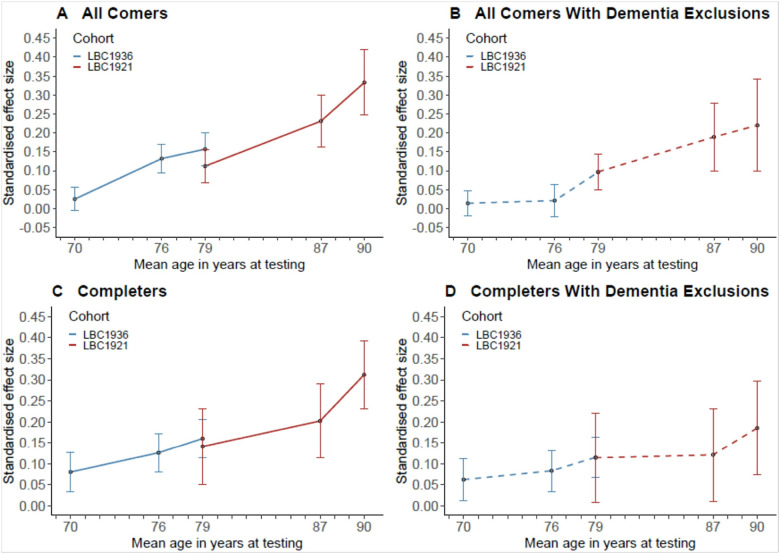
Standardised effect sizes for the associations of *APOE* e4 status on Moray House Test (MHT) score at each age in later life for the LBC1936 and the LBC1921. Solid lines (plots A and C) denote results for the full cohort (corresponding to Model 2 in Tables 3 and 4) and dashed lines (model B and D) denote results for the subsamples without dementia up to the end of the follow-up period (corresponding to Model 2 in Supplementary Tables S2 and S3). Note that both cohorts were dementia free at baseline. Effect sizes are presented as positive values for the purposes of showing the magnitude of associations between *APOE*e4 status and MHT sores at each age. Error bars represent standard errors.

Data were first analysed from all comers using regression models to test cross-sectional effects. There were significant effects of the e4 allele of *APOE* on Moray House Test scores at ages 76 and 79 in the LBC1936 and ages 79, 87, and 90 in the LBC1921 (all p values at or lower than 0.009) (Table 3). There was no significant effect at age 11 in either cohort nor at age 70 in the LBC1936. For all comers and adjusting for age, sex and health covariates (model 2), the absolute effect size (standardised beta) of e4 on MHT scores is as follows: 0.03 at 70 (this is the only non-significant result); 0.13 at 76; 0.13 at 79 (meta-analysis of LBC1921 and LBC1936); 0.23 at age 87; and 0.33 at age 90 (Table 3, Fig. 4). The pattern for completers is similar: 0.08 at 70 (non-significant); 0.13 at 76; 0.16 at 79 (meta-analysis of LBC1921 and LBC1936); 0.20 at age 87; and 0.31 at age 90 (Table 4, Fig. 4). After excluding those who subsequently developed dementia, the pattern is similar in the all-comers analysis (Supplementary Table 2, Fig. 4). However, for the completers most results become non-significant and the pattern is less clear; we note that the N is small at older ages in the LBC1921 (Supplementary Table 3, Fig. 4).

### Growth curve modelling: the APOE e4 effect sizes on intercepts (age 70 and 79 for LBC1936 and LBC1921, respectively) and slopes (age 70 to age 79, and age 79 to age 90)

**Figure 5:**
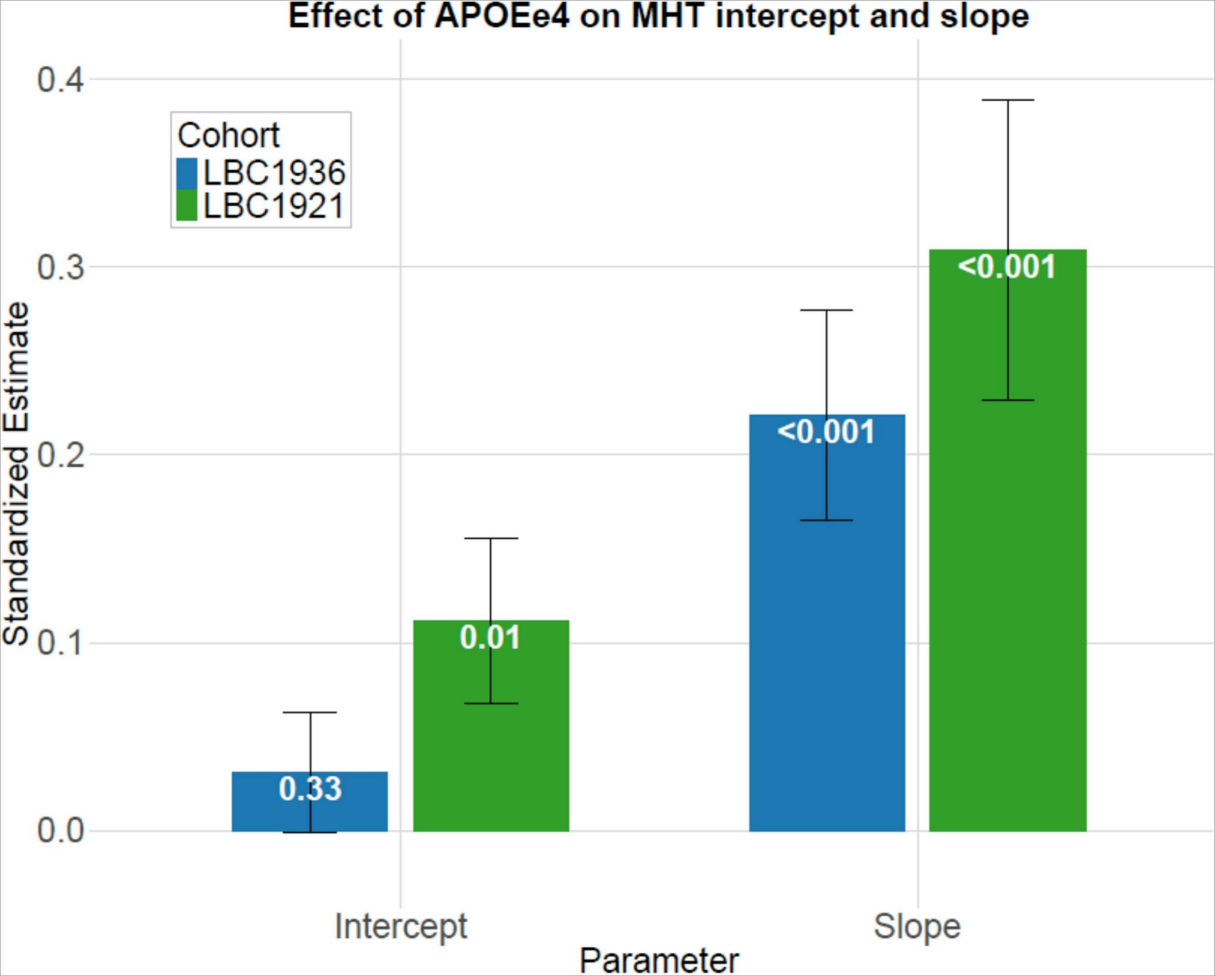
Standardised effect sizes (with standard error bars) for the effect of *APOE* e4 status on Moray House Test intercepts and slopes obtained using growth curve modelling. Values in white are P-values. Standardised effect sizes are derived from longitudinal growth curve models which have been adjusted for age, sex, CVD, stroke, diabetes, and hypertension (see Table 5).

We applied growth curve modelling to the Moray House Test data from LBC1936 and LBC1921. This affords an estimate of the effect size of the e4 *APOE* allele on the intercepts (age 70 and age 79, respectively, for the two cohorts) and the cognitive-change slopes (age 70 to 79 for LBC1936 and age 79 to 90 for the LBC1921). Age, sex, and health variables (cardiovascular disease, diabetes, stroke hypertension) were included as covariates. There was no significant effect of *APOE* e4 possession on the LBC1936 intercept (absolute standardised estimate = 0.03, p = 0.33), which accords with the cross-sectional analyses (Table 5, Fig. 5). There was a significant association between *APOE* e4 possession and steeper cognitive ageing in LBC1936 slope (age 70 to age 79); the absolute effect size was 0.22 (p < 0.001). In LBC1921, those who were carriers of the *APOE* e4 allele had lower MHT baseline scores at age 79 (intercept) and steeper declines (slope) in MHT scores from age 79 to 90. The *APOE* e4 effects for LBC1921 were: intercept effect size = 0.11 (p = 0.010); slope effect size = 0.31 (p < 0.001). Although the LBC1921 effect size is numerically larger than that of the LBC1936, their 95% confidence intervals overlap (Table 5).

### Component loadings of the Moray House Test

An analysis was carried out to show the reader the cognitive credentials of the Moray House Test as an indicator of general cognitive functioning *(g)* in older age. Principal components analysis was conducted on the Moray House Test and 13 other diverse cognitive tests administered to the Lothian Birth Cohort 1936 at mean age of about 70 (their Wave 1 in older age). This allows us to contextualise the MHT (a measure of general cognitive function designed over a century ago) within an extensive multi-domain cognitive battery of currently-used cognitive tests. The N was 930. The scree slope and Eigenvalues greater than 1 criterion indicated the presence of two components, though the first unrotated component was much larger than the second (Supplementary Information and Supplementary Fig. 1). The first unrotated component accounted for 39% of the total variance in the tests. The Moray House Test had the highest loading, at 0.83; all tests loaded at 0.4 or greater (Supplementary Fig. 2). The next four highest-loaded tests after Moray House Test were Wechsler Symbol Search (0.73; assessing the cognitive domain of processing speed), the average of National Adult Reading Test and Wechsler Test of Adult Reading (0.73; verbal knowledge/crystallised ability), Wechsler Matrix Reasoning (0.70; non-verbal reasoning), and Wechsler Block Design (0.69; spatial ability). Therefore, the general component, here, has a several high loadings from tests assessing diverse cognitive domains, and the Moray House Test has a very high loading on general cognitive ability.

## Discussion

The effect size of *APOE* e4 + status on general cognitive ability rises from about zero at age 70 (and age 11) to about a standardised effect of 0.3 at age 90. If one tends to think of genetic variation offering a static effect, *APOE* variation’s effect on cognitive function rebuts that (as do heritability results across childhood to adolescence (50)). A main novelty in the present study was the use of the same test in the same people from age 11 to age 90, albeit that that was achieved by bolting together two (very similar) cohorts at age 79. We found no differences between *APOE* e4 carriers and Moray House Test scores at ages 11 or 70; of course, that is a large age gap and we may not state that differences did not appear and then disappear during that epoch (29). The results agree with the Whitehall II study’s results on global cognitive function, i.e., that there is no difference between *APOE* e4 heterozygotes at about age 70 but the *APOE* e4 carriers score lower by age 75 and decline faster (29). It also accords with studies that indicate inconclusive effects of *APOE* e4 at younger ages on cognitive and brain-imaging markers (51, 52), although there is evidence for an effect of e4 (especially for e4e4 homozygotes) on poorer brain white matter health in middle to older aged people without dementia (53). The present study extends previous research with a validated test of general cognitive ability administered in youth and on six occasions from age 70 to 90. Growth curve modelling found that possession of at least one e4 allele of *APOE* was associated with cognitive slope (decline) in the LBC1921 (age 79 to 90) and 1936 (age 70 to 79). The effect size was numerically larger in the LBC1921, although the difference between the two betas was not significant; a larger N would be required to establish whether the slope is greater in the ninth versus eighth decades. We showed that the Moray House Test is strongly loaded on general cognitive ability which was our intended outcome construct.

The study meets recommendations (24) for this field of research by, e.g., having a large starting sample with a narrow age range, having a long follow-up period (including 20 years in older age), focussing on one cognitive phenotype that is affected by ageing and dementia, and trying to rule out dementia subsequent to the first wave of testing in older age (24). The latter was done by clinical assessment based on medical records and by some in-person visits. This was done to try to discover if all of the effect of *APOE* variation (here, possession of one or two e4 alleles) on individual differences in apparently healthy cognitive ageing was due to preclinical dementia; this has been referred to as the “prodromal effect” (24). The possibility that people with the e4 allele of *APOE* might have an “altered neural endophenotype” and, as a result of that, undergo steeper age-related cognitive decline, has been referred to as the “phenotype effect” (24). In the present study, for the phenotype that we studied, there is some evidence for both effects’ operating, though larger numbers of participants at the older ages are required more clearly to decide between these possibilities or to demonstrate that they are both operating.

The mechanistic trail from the current results should be interesting and practically valuable. It is important to discover what it is about possessing one or more *APOE* e4 alleles that results in steeper cognitive decline. There is much that is known about the biological actions of apolipoprotein E from work on humans and other research models, and there are hints at therapeutic approaches (54). Further human-based research could usefully search for brain-imaging-based variables that mediate the association between *APOE* variation and general cognitive functioning (51, 55), though these variables would need to be available nearer to the ages at which *APOE* variation has a significant effect on cognitive functioning. There is already a suggestion that brain white matter health might show increasing negative effects of *APOE* variation with older age and that this might occur at ages prior to the *APOE*-cognition associations (53). In addition to the increasing range of brain-imaging-based variables that are available, various ‘omics panels will offer clues to the ways by which *APOE* variation affects cognitive functioning (56).

This study has imitations. Here, we concentrated on providing a clean result for the effect of *APOE* e4 variation on general cognitive functioning in older age. A substantial review emphasised that research on *APOE*’*s* association with cognitive function should adopt a design and choice of cognitive phenotype that is appropriate to the neurobiological mechanism being investigated (24). We chose to investigate general/global cognitive functioning as it is the factor that accounts for much of the variance in age-associated cognitive decline across diverse cognitive domains and is affected in dementia (57–59). By using a single, well-validated cognitive test in two very similar and geographically co-located, narrow-age samples the results are relatively free from possible moderators of the effect. Of course, this means that there are other investigations that would be valuable to broaden the results. It will be useful to examine further: different cognitive domains (although much of the effect of ageing on cognitive capability is on general cognitive ability) (60); sex differences and geographically varied samples (61–63); and the possible moderating effect of medical conditions. It will also be useful to have longer follow-ups so that even older people may be included and later incident cases of dementia can be used for exclusion. Although, we should introduce some caution in being too conscientious here: that is, if the goal is to exclude those who were already on a dementia trajectory at study entry, then, say, 20 years of subsequent observation might be too long a window—one might be excluding cognitively healthy individuals who, much later, aged into dementia and who were not in a prodromal state when the *APOE* e4-cognition association was examined. A balance might be to exclude only those who developed dementia within a ‘preclinical window’ (e.g., within 5 to 10 years of baseline), as these cases are more likely to have had undetected pathology at age 70 or 79. We had insufficient power to include tests of other types of *APOE* variation, such as being homozygous for e4 and possession of one or more e2 alleles. Thus, compared with the Whitehall II study, owing to our later starting age of 70 in older age and a smaller sample size, we were not able to test their findings of specific effects of *APOE* e4 homozygotes and possibly better cognitive scores of *APOE* e4 heterozygotes between ages 45 and 55 years (29). Larger sample sizes—especially at the older ages covered herein—are needed formally to test for differences in the effect size of *APOE* e4 status on cognitive test scores between different ages. We note that our principal components analysis of the Moray House Test with 13 other tests had a strong first unrotated principal component (general cognitive ability). The presence of a small second component was probably a result of the battery of tests containing four assessments of processing speed.

## Concluding remarks

*APOE* variation is a relative rarity in cognitive ageing research in being a robust predictor of people’s differences in age-related cognitive decline. Here, we find that its effects are around null in childhood and at about age 70 and increase to a large effect (64) through the ninth decade. As is known from phenylketonuria, genetic variation is not destiny; therefore, research that explores the pathways through which *APOE* variation works could provide useful information toward alleviating some of the health inequalities in cognitive ageing.

## Figures and Tables

**Table 1:** Characteristics of participants at baseline in older-age by *APOE*e4 status

Characteristics	Lothian Birth Cohort 1936 (baseline: age 70 years)				Lothian Birth Cohort 1921 (baseline: age 79 years)			
		All	*APOE*e4 yes	*APOE*e4 no		All	*APOE*e4 yes	*APOE*e4 no
	N	M (SD) or N (%)			N	M (SD) or N (%)		
**All comers, N (%)**	**1010**		300 (30%)	710 (70%)	**539**		143 (27%)	396 (73%)
Age	1010	69.5 (0.8)	69.5 (0.8)	69.5 (0.8)	539	79.1 (0.6)	79.0 (0.6)	79.1 (0.6)
Sex, female	1010	508 (50%)	142 (28%)	366 (72%)	539	312 (58%)	63 (20%)	158 (80%)
History of hypertension	1010	407 (40%)	116 (29%)	291 (71%)	537	221 (41%)	63 (29%)	158 (71%)
History of diabetes	1010	84 (8%)	19 (23%)	65 (77%)	539	27 (5%)	4 (15%)	23 (85%)
History of CVD	1010	247 (24%)	80 (32%)	167 (68%)	533	159 (29%)	47 (30%)	112 (70%)
History of stroke	1010	52 (5%)	11 (21%)	41 (79%)	539	45 (8%)	14 (31%)	31 (69%)
* **Completers, N (%)**	**457**		137 (30%)	320 (79%)	**119**		25 (21%)	94 (79%)
Age	457	69.5 (0.8)	69.6 (0.9)	69.5 (0.8)	119	79.1 (0.5)	79.0 (0.6)	79.1 (0.5)
Sex, female	457	229 (50%)	57 (25%)	172 (75%)	119	67 (56%)	11 (16%)	56 (84%)
History of hypertension	457	162 (35%)	46 (28%)	116 (72%)	118	40 (34%)	8 (20%)	32 (80%)
History of diabetes	457	27 (6%)	10 (37%)	17 (63%)	119	3 (3%)	0 (0%)	3 (100)
History of CVD	457	102 (22%)	29 (28%)	73 (72%)	117	30 (26%)	5 (17%)	25 (83%)
History of stroke	457	14 (3%)	4 (29%)	10 (71%))	119	9 (8%)	1 (11%)	8 (89%)

Note: CVD = cardiovascular disease.

* Completers are participants who attended all three waves of testing in older age (LBC1936: ages 70, 76, 79; LBC1921: ages 79, 87, 90) and completed the Moray House Test during each of these waves.

**Table 2:** Moray House Test (MHT) scores at each time-point according to *APOE e4* status, for all comers and completers

	Lothian Birth Cohort 1936							
	All comers				*Completers			
		All	*APOE*e4 yes	*APOE*e4 no		All	*APOE*e4 yes	*APOE*e4 no
	N	M (SD)			N	M (SD)		
MHT at age 11 (childhood)	954	49.3 (11.3)	49.3 (115)	49.3 (11.2)	430	51.6 (11.0)	50.7 (12.1)	51.9 (10.4)
MHT at age 70 (wave 1)	1001	64.6 (8.0)	64.3 (8.2)	64.7 (8.0)	457	66.7 (6.8)	65.8 (7.7)	67.0 (6.4)
MHT at age 76 (wave 3)	636	63.0 (9.5)	61.0 (10.7)	63.9 (8.9)	457	64.5 (8.6)	62.8 (9.6)	65.3 (7.9)
MHT at age 79 (wave 4)	471	62.7 (9.8)	60.2 (10.3)	63.7 (9.5)	457	63.0 (9.6)	60.6 (10.1)	64.0 (9.2)
	Lothian Birth Cohort 1921							
	All comers				*Completers			
MHT at age 11 (childhood)	483	46.6 (11.8)	45.8 (11.2)	46.9 (12.1)	104	49.2 (11.0)	45.7 (10.8)	50.1 (10.9)
MHT at age 79 (wave 1)	533	59.5 (10.4)	57.5 (10.7)	60.2 (10.3)	119	63.4 (8.2)	61.4 (7.6)	63.9 (8.3)
MHT at age 87 (wave 3)	198	54.2 (13.8)	48.8 (15.9)	55.8 (12.8)	119	58.0 (10.9)	53.8 (11.3)	59.1 (10.6)
MHT at age 90 (wave 4)	120	51.7 (14.5)	44.0 (14.0)	53.7 (14.1)	119	51.9 (14.4)	44.0 (14.0)	54.1 (13.8)

Note:

* Completers are participants who attended all three waves of testing in older age (LBC1936: ages 70, 76, 79; LBC1921: ages 79, 87, 90) and completed the MHT during each of these waves.

For the LBC1936, exact ages in years (M ± SD) were as follows: age 11 = 10.9 ± 0.3; age 70 = 69.5 ± 0.8; age 76 = 76.2 ± 0.7; age 79 = 79.3 ± 0.6.

For the LBC1921, exact ages in years (M ± SD) were as follows: age 11 = 10.9 ± 0.3; age 79 = 79.5 ± 0.7; age 87 = 86.6 ± 0.4; age 90 = 90.1 ± 0.1.

**Table 3:** Main regression models for all comers: cross-sectional associations between *APOE* e4 status and Moray House Test (MHT) scores at each age separately

		Lothian Birth Cohort 1936 (N = 1010 at baseline age 70)								
		Model 1 – age + sex					Model 2 – age + sex + health covariates			
MHT	N	Std est	SE	P value	95% CI	N	Std est	SE	P value	95% CI
Age 11	954	0.005	0.032	0.884	−0.058, 0.067					
Age 70	^1^1001	−0.022	0.031	0.483	−0.083, 0.039	1001	−0.025	0.031	0.415	−0.086, 0.036
Age 76	636	−0.132	0.038	**0.001**	−0.207, −0.056	634	−0.132	0.038	**0.001**	−0.208, −0.057
Age 79	471	−0.161	0.044	**< 0.001**	−0.248, −0.074	468	−0.157*	0.044	**< 0.001**	−0.244, −0.070
		Lothian Birth Cohort 1921 (N = 539 at baseline age 79)								
Age 11	483	−0.046	0.044	0.302	−0.153, 0.041					
Age 79	^2^533	−0.114	0.043	**0.008**	−0.197, −0.030	525	−0.112*	0.043	**0.009**	−0.196, −0.029
Age 87	198	−0.224	0.070	**0.001**	−0.362, −0.086	196	−0.231	0.069	**0.001**	−0.367, −0.095
Age 90	120	−0.296	0.088	**0.001**	−0.469, −0.123	120	−0.333	0.086	**< 0.001**	−0.502, −0.165

Note:

1 In the LBC1936, N = 9 were missing a MHT score at older-age baseline age 70;

2 In the LBC1921, N = 6 were missing an MHT score at older-age baseline age 79. The health covariates (yes/no) in model 2 are CVD, diabetes, stroke, and hypertension.

* The meta-analytic effect size across both cohorts at age 79 (adjusted model 2—see asterisked values above) is −0.134; SE = 0.031; P < 0.0001; 95%CI = −0.194, −0.074.

**Table 4:** Main regression models for completers only: cross-sectional associations between *APOE* e4 status and Moray House Test (MHT) scores at each age separately

	Lothian Birth Cohort 1936 (N = 457)									
		Model 1 – age + sex					Model 2 – age + sex + health covariates			
MHT	N	Std est	SE	P value	95% CI	N	Std est	SE	P value	95% CI
Age 11	430	−0.046	0.047	0.330	−0.139, 0.047					
Age 70	457	−0.080	0.046	0.081	−0.171, 0.010	457	−0.080	0.046	0.082	−0.172, 0.010
Age 76	457	−0.126	0.046	**0.006**	−0.216, −0.036	457	−0.126	0.046	**0.006**	−0.215, −0.036
Age 79	457	−0.161	0.045	**< 0.001**	−0.249, −0.073	454	−0.160*	0.045	**< 0.001**	−0.248, −0.071
	Lothian Birth Cohort 1921 (N = 119)									
Age 11	104	−0.112	0.093	0.232	−0.294, 0.011					
Age 79	119	−0.134	0.090	0.137	−0.311, 0.043	116	−0.141*	0.090	0.119	−0.318, −0.036
Age 87	119	−0.202	0.088	**0.021**	−0.374, −0.031	119	−0.202	0.088	**0.022**	−0.376, −0.029
Age 90	119	−0.289	0.082	**< 0.001**	−0.451, −0.128	119	−0.312	0.081	**< 0.001**	−0.471, −0.153

Note: Completers are those who attended, and sat the MHT, at all 3 waves in later life. The health covariates in model 2 are CVD, diabetes, stroke, and hypertension (yes/no).

* The meta-analytic effect size across both cohorts at age 79 (adjusted model 2–see asterisked values above) is -−0.156; SE = 0.040; P = 0.0001; 95%CI = −0.235, −0.077.

**Table 5:** Longitudinal growth models: associations of *APOE* e4 with change in Moray House Test (MHT) score across later life (fully-adjusted)

	Lothian Birth Cohort 1936 (N = 1010 at baseline)							
	Intercept (*APOE* e4 and MHT at baseline, age 70)				Slope (*APOE* e4 and MHT change from age 70 to 79)			
Predictors	Std est	SE	P value	95% CI	Std est	SE	P value	95% CI
*APOE* e4	−0.031	0.032	0.331	−0.094, 0.032	−0.221	0.056	**< 0.001**	−0.330, −0.112
CFI	1.00							
RMSEA	0.00							
SRMR	0.00							
	Lothian Birth Cohort 1921 (N = 539 at baseline)							
	Intercept (APOE e4 and MHT at baseline, age 79)				Slope (APOE e4 and MHT change from age 79 to 90)			
*APOE* e4	−0.112	0.044	**0.010**	−0.197, −0.027	−0.309	0.080	**< 0.001**	−0.467, −0.151
CFI	0.95							
RMSEA	0.06							
SRMR	0.03							

Note: The growth curve model for each cohort is fully-adjusted for all wave 1 covariates: age; sex; CVD; stroke; high blood pressure; diabetes. Slope values are based on change in Moray House Test score from age 70 to age 79 (for LBC1936) and from age 79 to age 90 (for LBC1921). Model fit was tested using absolute fit indices: Comparative Fit Index (CFI; values > 0.95 considered acceptable), Root Mean Square Error of Approximation (RMSEA; values < 0.06 considered acceptable), and Standardized Root Mean Square Residual (SRMR; values < 0.08 considered acceptable).
